# Mitochondrial density and cell area changes in the ciliate *Paramecium bursaria* under constant darkness: effects of symbiotic *Chlorella variabilis* and nutrient availability

**DOI:** 10.1038/s41598-026-41878-5

**Published:** 2026-02-27

**Authors:** Shingo Asari, Yuuki Kodama

**Affiliations:** 1 Department of Life Sciences, Faculty of Life and Environmental Sciences, Shimane University, Matsue-shi, Japan; 2 Institute of Agricultural and Life Sciences, Academic Assembly, Shimane University, Matsue-shi, Japan

**Keywords:** *Chlorella variabilis*, Endosymbiosis, Mitochondria, *Paramecium bursaria*, Symbiotic algae, Ecology, Ecology, Microbiology, Plant sciences

## Abstract

*Paramecium bursaria* and its symbiotic association with *Chlorella variabilis* influence host organelles. Previous studies have reported reduced mitochondria and trichocysts in algae-bearing *P. bursaria* cells, suggesting that the digestion of symbiotic algae may provide nutrients for trichocyst synthesis. However, the response of host mitochondria to symbiont loss under prolonged darkness remains unclear. Here, we examined the mitochondrial dynamics and cell morphology in algae-bearing and alga-free *P. bursaria* under constant darkness combined with feeding or starvation. Algal reduction was quantified using differential interference contrast image intensity, and host mitochondria were visualized using MitoBright LT Green. Under dark conditions with starvation, symbiotic algae and cell area decreased markedly, whereas mitochondrial fluorescence remained largely unchanged in algae-bearing cells. Gradual loss of algae despite feeding preserved both cell area and mitochondrial density. In alga-free cells, starvation caused early mitochondrial decline, followed by partial recovery, whereas feeding supported maintenance or enhancement. These findings indicate that mitochondrial density does not increase as algae decrease; instead, nutrient availability is critical for sustaining mitochondria in prolonged darkness. Our results provide insights into organelle-level responses to symbiont loss and the mechanisms underlying endosymbiotic resilience under environmental stress, with implications for mutualistic stability in changing ecosystems.

**Supplementary Information:**

The online version contains supplementary material available at 10.1038/s41598-026-41878-5.

## Introduction

*Paramecium bursaria* is a ciliate protist that is approximately 100–150 μm long and can symbiotically host approximately 700 *Chlorella variabilis* cells in its cytoplasm. The relationship between symbiotic *Chlorella* spp. and the host *P. bursaria* is mutualism, with the symbiotic algae providing the host with photosynthetic oxygen and sugars, such as maltose^1,2^, and *P. bursaria* providing the algae with nitrogen sources and carbon dioxide^3–6^. This mutualism allows *P. bursaria* to survive for long periods under starvation conditions if light is available^7^ and protects it from yeast and bacterial infections^8^. Despite this mutualism, both alga-free *P. bursaria*, in which symbiotic algae have been removed, and *Chlorella* spp. isolated from algae-bearing *P. bursaria* can grow independently of each other^9^. Methods for removing symbiotic algae from algae-bearing *P. bursaria* include inducing the host *P. bursaria* to multiply rapidly in a nutritive medium, such as an algal-bacterial medium^10^, and keeping algae-bearing *P. bursaria* in the dark^7,11,12^. When algae-bearing *P. bursaria* are mass cultured in flasks at 25 °C and constant darkness (DD) for 2 to 3 weeks, and only the *P. bursaria* cells are allowed to divide without growing algae, they become alga-free white *P. bursaria* cells^13^. Alga-free *P. bursaria* cells can also be obtained by treatment with X-irradiation^14^, the photosynthesis inhibitor DCMU^5^, the herbicide paraquat^15^, acrylamide^16^, and the protein synthesis inhibitor cycloheximide in eukaryotes^17^. The symbiotic *Chlorella* spp. isolated from algae-bearing *P. bursaria* can also be cultured outside their hosts^18^. Furthermore, when both cultured alone are mixed, the algae are ingested by the alga-free *P. bursaria* cells, and some of the algae in the *P. bursaria* digestive vacuole (DV) can escape digestion, and re-symbiosis can be established^9,19^. Thus, alga-free *P. bursaria* can be easily obtained through various methods and re-infected with the symbiotic algae. This allows researchers to study the establishment and maintenance mechanisms of endosymbiosis under controlled laboratory conditions. Therefore, the endosymbiotic relationship between *P. bursaria* and *Chlorella* spp. is a model system for studying secondary symbiosis and host-symbiont interactions^9,20,21^.

The presence of symbiotic algae affects various host phenotypes, including a reduction in the number of host trichocysts^22,23^, mitochondria^24^, and cytoplasmic crystals^25^, a decrease in total cell protein content^24^, enhanced heat tolerance^26,27^, and influences photoaccumulation and circadian rhythms^27–31^. Symbiotic algae are individually enclosed in the perialgal vacuole (PV) membrane which is a type of symbiosome membrane and positioned below the cortex of *P. bursaria*, where host mitochondria and trichocysts are present^19,23,24,32,33^. In a previous study, the effect of symbiotic algae on the trichocysts of the host under starvation conditions was examined. Using indirect immunofluorescence microscopy with a monoclonal antibody specific for trichocysts, it was shown that trichocysts in alga-free *P. bursaria* degrade faster under starvation than those in algae-bearing ones^34^. Trichocysts are the defensive extrusomes of *Paramecium *spp.^35^. It has been suggested that the digestion of symbiotic algae provides nutrition that promotes trichocyst synthesis, allowing the host to survive for a longer time under nutrient-poor conditions^34^. The PV membrane is known to be closely associated with the symbiotic algal cell wall and connects to the host mitochondria and ER, forming a network of connections. Recently, symbiotic *C. variabilis* inside the PV of the ciliate *Stentor pyriformis* was found to be interspersed with mitochondria at the cell surface^36^. This suggests that intimate connections facilitate material exchange between the host and symbiont^37^. These studies suggest a possible negative correlation between the density of symbiotic *Chlorella* spp. and the density of mitochondria in the host *P. bursaria*.

However, whether host mitochondria respond to symbiont loss under prolonged darkness and how nutrient availability modulates this response remain unknown. This gap limits our understanding of organelle-level adaptations in endosymbiotic systems under environmental stress. Addressing this question is essential for clarifying how mutualistic associations persist under changing environmental conditions.

In this study, we artificially reduced the density of symbiotic *Chlorella* sp. in algae-bearing *P. bursaria* under DD conditions and examined how mitochondrial density and cell area respond to feeding and starvation in both algae-bearing and alga-free cells. Algal reduction was quantified by the mean differential interference contrast (DIC) image intensity, and host mitochondria were visualized using MitoBright LT Green (MitoBright). We also measured the cell area as an indicator of the nutritional status.

## Materials and methods

### Strains and cultures of *P. bursaria* cells

Two *P. bursaria* strains, symbiotic alga-free Yad1w (syngen B3 or R3, mating type I^38^) and algae-bearing Yad1g1N (syngen B1 or R3, mating type I^38^), were used in this study. The culture medium, prepared using red pea (*Pisum sativum*)^39^ extract in modified Dryl’s solution (MDS) (NaH₂PO₄·2H₂O replaced with KH₂PO₄)^40^, was inoculated with non-pathogenic *Klebsiella aerogenes* (ATCC35028) 1 d before use^41^. The strain Yad1w was derived from the *Chlorella*-bearing *P. bursaria* strain Yad1g by repeated cloning and cultivation under dark conditions^42^. The strain Yad1g1N was produced from Yad1w cells by infection with cloned symbiotic *C. variabilis* strain 1 N cells^23^.

### Cultivation of alga-free and algae-bearing *P. bursaria* cells

*P. bursaria* cells were divided into two groups, one fed (i.e., feeding) and one not fed (i.e., starvation), and they were incubated in an incubator at 22 ± 1 °C in constant darkness (DD). The feeding group received 2 ml of culture medium every 24–48 h. The day the cells were placed in an incubator was considered day 0 of the experiment. Algae-bearing *P. bursaria* cells were incubated for 30 or 35 d in the feeding group and for 15 or 24 d in the starvation group. Alga-free *P. bursaria* cells were incubated for 30 d in the feeding group and for 17 d in the starvation group.

### Observation of symbiotic *Chlorella* sp. reduction and changes in host cell area

**Fig. 1:**
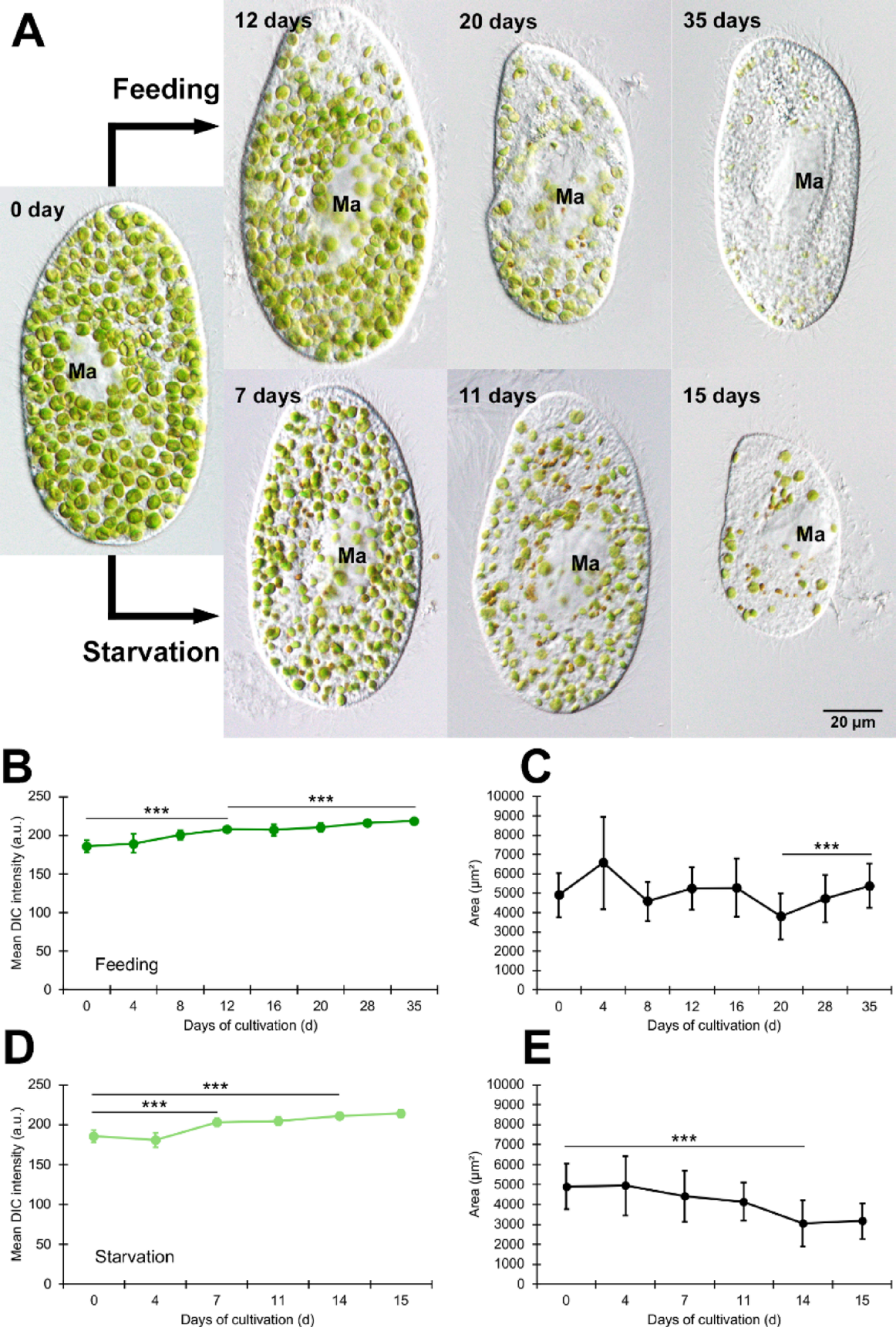
Changes in symbiotic algal density and host cell area in algae-bearing *P. bursaria* under constant darkness (DD). **(A)** Representative DIC micrographs: day 0 (middle panel), DD/feeding (upper panels), and DD/starvation (lower panels). Scale bar = 20 μm. (B–E) Quantitative analyses corresponding to panel A: **(B)** Mean DIC image intensity (a.u.) under feeding; lower values indicate higher algal densities. **(C)** Mean cell area (µm²) under feeding conditions. **(D)** Mean DIC image intensity (a.u.) under starvation. **(E)** Mean cell area (µm²) under starvation. Error bars = mean ± SD. The raw data used to generate Fig. 1 are provided in Supplementary S1, and the complete statistics are provided in Supplementary S4. Asterisks indicate Bonferroni-adjusted significance:  *P <* 0.001 (***).

Brightness (mean DIC image intensity, a.u.) was used as an inverse proxy for algal abundance (brighter cytoplasm = fewer algae). Direct counting of algae was impractical because each cell can contain up to approximately1,000 algae, and pixel-based quantification is not applicable to DIC images. In a 1.5 ml microtube, 500 µl of algae-bearing *P. bursaria* cells cultured under DD/feeding or starvation conditions were added, and 500 µl of 8% paraformaldehyde (PFA) was mixed for fixation. The time points for comparison were selected to represent the stages across the experimental period, where morphological changes were most pronounced. The fixed cells were observed under a DIC microscope (BX51; Olympus, Tokyo, Japan), and micrographs were obtained using a digital camera for microscopy (DP73; Olympus, Tokyo, Japan). The camera exposure (shutter) time was kept within a fixed window of 1.6–2.2 ms, with identical gain and binning settings across the time-course acquisitions. To quantitatively determine the density of symbiotic algae, DIC micrographs were analyzed for the mean DIC image intensity using CellSens Dimension software (Olympus, Tokyo, Japan). The mean DIC image intensity (a.u.) was used for these analyses. The mean DIC image intensity is already area-normalized. Simultaneously, the cell area of *P. bursaria* cells was measured, and the average cell area was calculated. Ten to 20 cells were analyzed per day in both the feeding and starvation groups. All images were captured during the same time window each day, between 9:00 and 12:00 a.m., to ensure consistency across conditions. Figures 1C and E and 3C and E were derived from separate experimental sets, each conducted under the same DD conditions but analyzed independently.

### Visualization of *P. bursaria* mitochondria with MitoBright

Twenty to forty milliliters of algae-bearing or alga-free *P. bursaria* was strained through two layers of KimWipes to remove gross debris and subsequently filtered using a plastic beaker equipped with a 15 μm pore nylon mesh. The cells were harvested using a hand-operated centrifuge (UKG-2; Uchida Rikakiki, Tokyo, Japan) and adjusted to 1 ml. Approximately 80, 000–100, 000 *P. bursaria* cells were present in this suspension. Nickel chloride (NiCl_2_) and potassium iodide (KI) have been reported to inhibit ciliary motion in *Tetrahymena* and *Paramecium* by inhibiting axonemal dynein^43,44^. To minimize non-mitochondrial fluorescence from dye uptake into DVs, ciliary motion was inhibited with 0.1 mM NiCl₂ prior to dye exposure, followed by washing and rapid imaging of the cells. A reversed order (dye first, NiCl₂ second) produced DV-associated-fluorescence. This treatment immediately inhibited ciliary movement in *P. bursaria.*

MitoBright was purchased from Dojindo (Kumamoto, Japan). To stain the mitochondria with MitoBright, the 0.1 mmol/l MitoBright solution was diluted with *P. bursaria* cells suspension to prepare a 0.1 µmol/l MitoBright working solution as refer to the manufacturer’s manual (https://www.dojindo.com/manual/MT10_MT11_MT12/).

The *P. bursaria* cell suspension was incubated under constant dark conditions at 23 ± 1 °C for 3–5 min, and the cells were observed using a fluorescence microscope (BX53; Olympus, Tokyo, Japan) equipped with an Olympus fluorescence mirror unit U-FBNA (excitation 470–495 nm, emission 510–550 nm). The time points for comparison were selected to represent the stages across the experimental period, where morphological changes were most pronounced. Images were acquired using an Olympus DP74 system with exposures of 25–30 ms. Within each experimental series, the exposure, gain, and binning were kept constant. Histograms were examined to ensure that no pixels were saturated, and frames with clipping were excluded from quantification. MitoBright fluorescence was analyzed using Olympus cellSens Dimension software, and the mean fluorescence intensity (a.u.) was used for mitochondrial fluorescence. The mean fluorescence intensity is already area-normalized. All images were captured during the same time window each day, between 9:00 and 12:00 a.m., to ensure consistency across conditions. MitoBright fluorescence was used as a structural marker. Although mitochondrial density was defined as the area fraction of MitoBright-positive regions, all statistical analyses were based on the mean fluorescence intensity (a.u.).

### Statistical analysis

Each experiment was independently repeated three times under the same conditions to confirm reproducibility. Statistical analyses were performed on the pooled data from these replicates. Non-data rows (e.g., “n”, “Minimum”, “Average”, “SD”, “Maximum”) were excluded from the source spreadsheet. For each condition (Feeding or Starvation), observations were grouped by day and paired by replicate ID prior to statistical analysis. Normality at each time point was assessed using the Shapiro–Wilk test; when normality was satisfied, homogeneity of variances was assessed using Levene’s test. When both assumptions were met, we applied one-way ANOVA and reported partial η²; otherwise, we used the Kruskal–Wallis test and reported ε². Post hoc pairwise comparisons were performed only when the omnibus test was significant, using Welch’s t tests (for ANOVA) or Mann–Whitney U tests (for Kruskal–Wallis), with Bonferroni correction applied to two-sided p values (α = 0.05). The analyzed time points and sample sizes (n) are provided in the figure legends and in Supplementary file S4 (Sheets “SampleSizes” and “Pairwise”). Where omnibus tests were non-significant, no post hoc results were reported, and the interpretation was tempered accordingly. Analyses were performed in R (version 4.5.2)^45^ on macOS using the following packages: stats (Shapiro–Wilk, ANOVA, Welch’s t tests, Kruskal–Wallis), car (Levene’s test), rstatix and effectsize (effect sizes: partial η² for ANOVA; ε² computed as (H − k + 1)/(n − k) for Kruskal–Wallis), dplyr (data manipulation), readxl (Excel data import), and writexl (Excel export). Multiple-comparison correction used base R p.adjust with the Bonferroni correction method. Final figures were prepared in Microsoft Excel after statistical analysis in R.

## Results

### Changes in the density of symbiotic algae and in host cell area of algae-bearing *P. bursaria* under DD conditions culture

Figure 1A shows representative DIC micrographs, and panels B–E provide quantitative analyses corresponding to these images. On day 0, many green symbiotic algae were observed. In the feeding group (Fig. 1A, upper panels), numerous cells exhibiting reduced symbiotic algae from the head were observed on day 12. In contrast, in the starvation group (Fig. 1A, lower panels), a significant number of *P. bursaria* cells with reduced symbiotic algae were observed on day 7. Regarding cell area (µm²), prior to cultivation under DD conditions, most cells exhibited a large cell area. Cell area reduction occurred later under feeding than under starvation, indicating that nutrient availability delays cell shrinkage.

The changes in symbiotic algal density in the feeding group showed in Fig. 1A (upper panels) were represented by mean DIC image intensity (a.u.) (Fig. 1B). As illustrated in Fig. 1A, when the density of algae decreased, the area of the host cytoplasm, which appeared bright under DIC microscopy, increased. Consequently, as shown in Fig. 1B, a lower mean DIC intensity indicates a higher abundance of symbiotic algae, whereas a higher mean DIC intensity indicates fewer symbiotic algae. An omnibus Kruskal–Wallis test indicated significant differences across time points. Post hoc tests indicated that the DIC intensity increased significantly from day 0 to day 12 and further to day 35, reflecting progressive algal loss. For the analysis, DIC microscopy image data were used: 11 cells on day 0, 17 cells on day 4, 17 cells on day 8, 16 cells on day 12, 23 cells on day 16, 19 cells on day 20, 19 cells on day 28, and 20 cells on day 35 of the experiment.

Changes in the average cell area (µm²) in the feeding group are shown in Fig. 1C. Kruskal–Wallis tests showed significant differences, with day 20 being significantly smaller than day 35. The cell counts (n) used in Fig. 1C are identical to those in Fig. 1B.

Changes in the density of symbiotic algae in the starvation group (Fig. 1A, lower panels) were quantified using the mean DIC image intensity (a.u.) (Fig. 1D). The intensity increased after day 7, indicating a decrease in the density of the symbiotic algae. One-way- ANOVA (omnibus) indicated significant differences across time points; Bonferroni-adjusted post hoc t-tests confirmed significance between days 0 and 7 and between days 0 and 14. For the analysis, DIC microscopy image data were used: 11 cells on day 0, 16 cells on day 4, 19 cells on day 7, 16 cells on day 11, 16 cells on day 14, and 9 cells on day 15.

The change in the mean cell area (µm²) in the starvation group is shown in Fig. 1E. The mean cell area was significantly reduced on day 14. The Kruskal–Wallis omnibus test indicated significant differences, and Bonferroni-adjusted post hoc tests confirmed that day 14 was significantly smaller than day 0 (and smaller than days 4 and 7). The cell counts (n) used in Fig. 1E are identical to those in Fig. 1D.

Conclusions were based on the quantified means across biological replicates (Fig. 1B–E).

Under starvation conditions, cell death is expected. Although we did not perform systematic cell counting, our observations indicated that the number of surviving cells gradually decreased over time, and by the final day, approximately10% of the initial population remained. The images in Fig. 1A represent the typical morphology of surviving *P. bursaria* cells, which also exhibited a marked reduction in the density and size of symbiotic algae.

### MitoBright imaging of host mitochondrial localization in algae-bearing and alga-free *P. bursaria*

**Fig. 2:**
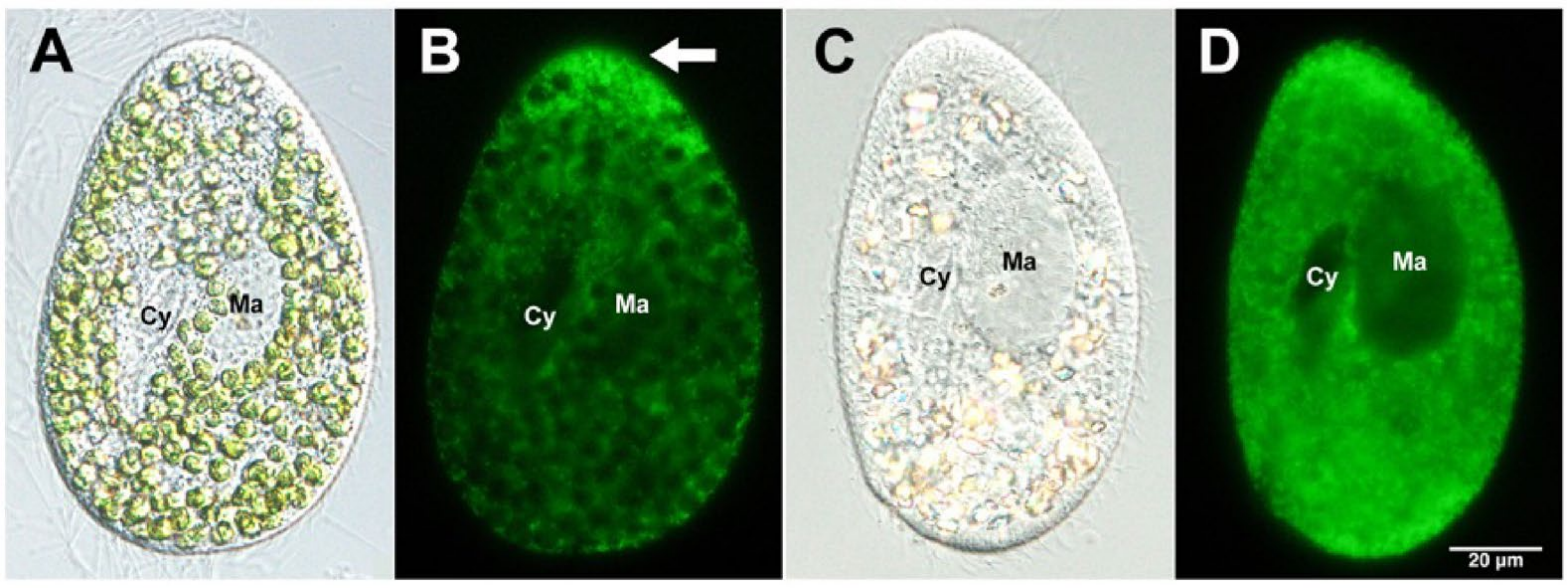
Representative fluorescence images of host mitochondria stained with MitoBright **(A)** DIC image of algae-bearing *P. bursaria*. **(B)** Corresponding fluorescence image showing an intense anterior signal (white arrow). **(C)** DIC image of the alga-free *P. bursaria*. **(D)** Corresponding fluorescence image showing broad mitochondrial distribution, excluding the macronucleus (Ma) and cytopharynx (Cy). Scale bar = 20 μm.

Fluorescence microscopy images of the host mitochondria stained with MitoBright are shown in Fig. 2. Mitochondria were observed as green fluorescence in both algae-bearing (Figs. 2A and B) and alga-free (Figs. 2C and D) *P. bursaria*. In algae-bearing *P. bursaria*, intense fluorescence was observed at the anterior end (Fig. 2B, white arrow). In regions where symbiotic algae were distributed in *P. bursaria*, weak fluorescence of the host mitochondria was observed surrounding the symbiotic algae (Figs. 2A and B). In the alga-free *P. bursaria*, host mitochondrial fluorescence was observed throughout the cell, excluding the macronucleus (Ma) and cytopharynx (Cy) (Figs. 2C and D).

### Changes in mitochondria and cell area of algae-bearing *P. bursaria* under DD conditions culture

**Fig. 3:**
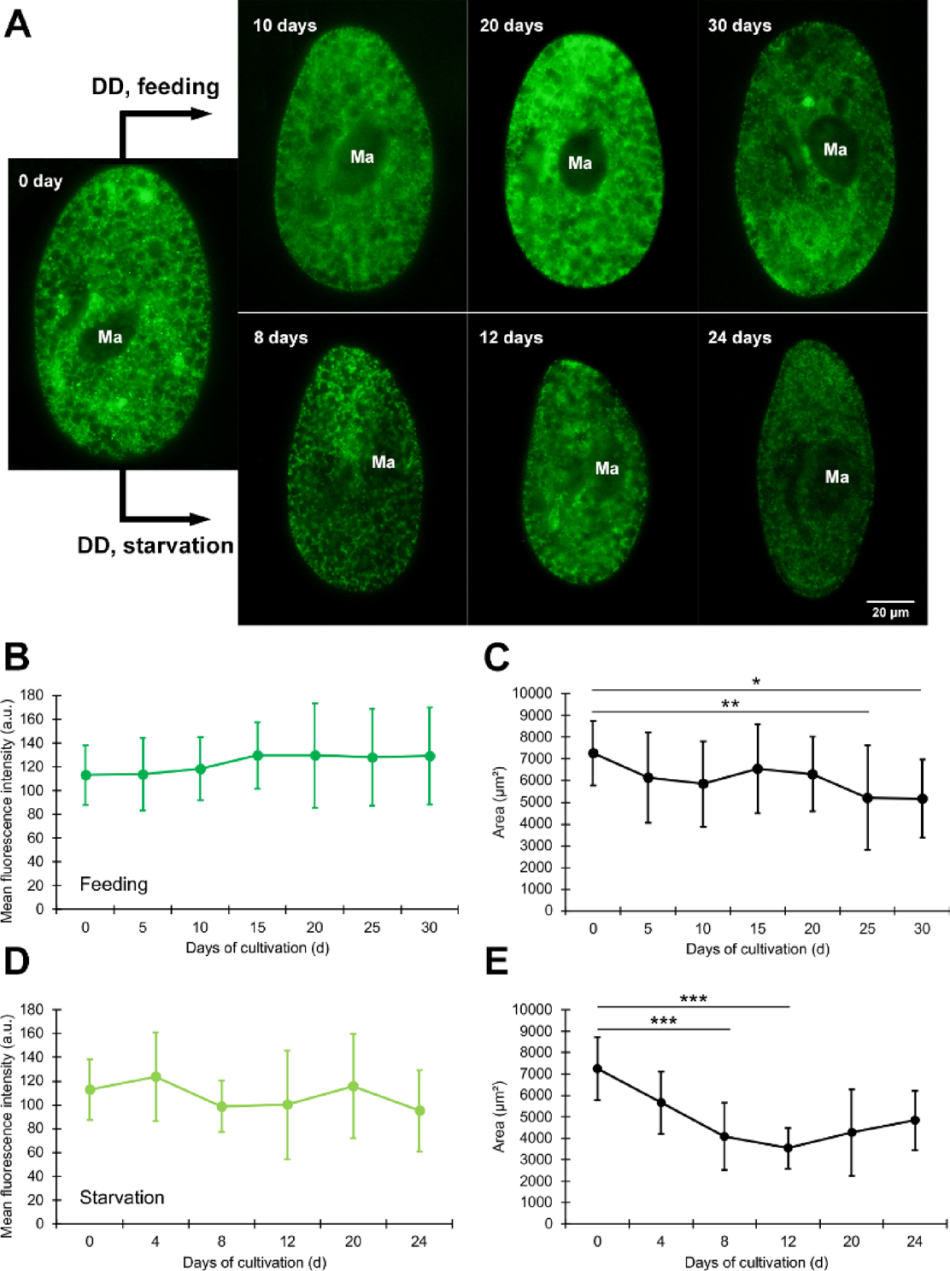
Host mitochondrial fluorescence and cell area in algae-bearing *P. bursaria* under constant darkness (DD) conditions. **(A)** Representative fluorescence micrographs: day 0 (middle panel), DD/feeding (upper panels), and DD/starvation (lower panels). Scale bar = 20 μm. **(B–E)** Quantitative analyses: (**B**) Mean mitochondrial fluorescence intensity (a.u.) under feeding. **(C)** Mean cell area (µm²) under feeding conditions. **(D)** Mean mitochondrial fluorescence intensity (a.u.) under starvation. **(E)** Mean cell area (µm²) under starvation. Error bars = mean ± SD. The raw data used to generate Fig. 3 are provided in Supplementary S2, and the complete statistics are provided in Supplementary S4. Asterisks indicate Bonferroni-adjusted- significance: *P* < 0.05 (*), *P* < 0.01 (**), and *P <* 0.001 (***).

Figure 3A shows representative fluorescence micrographs, and panels B–E provide quantitative analyses corresponding to these images. Figure 3A shows a micrograph depicting the mitochondrial fluorescence of algae-bearing *P. bursaria* under DD conditions. In the feeding group (Fig. 3A, upper panels), no significant alterations in mitochondrial fluorescence were observed on days 10, 20, and 30 after the initiation of DD incubation. In the starvation group (Fig. 3A, lower panels), also mitochondrial fluorescence did not differ significantly.

The mean fluorescence intensity (a.u.) of the host mitochondria in the algae-bearing *P. bursaria* in the feeding group is shown in Fig. 3B. A higher mean intensity indicates greater mitochondrial density. The mean fluorescence was 112.90 on day 0 of incubation under DD conditions and tended to increase from day 15 onwards. However, in the omnibus Kruskal–Wallis test, we found no statistically significant differences across time points; accordingly, no Bonferroni-adjusted post hoc tests were conducted. Fluorescence microscopy images of 22 cells on day 0, 15 cells on day 5, 15 cells on day 10, 20 cells on day 15, 26 cells on day 20, 19 cells on day 25, and 24 cells on day 30 were analyzed.

The mean cell area (µm²) in the feeding is shown in Fig. 3C. The host cell area decreased over time. The Kruskal–Wallis omnibus test was significant, and Bonferroni-adjusted post hoc tests identified days 0 vs. 25 and 0 vs. 30 as significant. The cell counts (n) used in Fig. 3C are identical to those in Fig. 3B.

The mean fluorescence intensity (a.u.) of the host mitochondria in the starvation group of algae-bearing *P. bursaria* is shown (Fig. 3D). In the Kruskal–Wallis omnibus test, time-course differences in mitochondrial fluorescence were not statistically significant; therefore, no post hoc tests were performed. Fluorescence microscopy images of 22 cells on day 0, 14 cells on day 4, 17 cells on day 8, 16 cells on day 12, 12 cells on day 20, and 10 cells on day 24 were analyzed.

The mean cell area (µm²) in the feeding is shown in Fig. 3E. The mean cell area decreased from day 4, reached a minimum within the experimental time course on day 12, and increased from day 20. The Kruskal–Wallis omnibus test was significant; Bonferroni-adjusted post hoc tests confirmed days 0 vs. 8 and 0 vs. 12 as significant. The cell counts (n) used in Fig. 3E are identical to those in Fig. 3D.

Conclusions were based on the quantified means across biological replicates (Fig. 3B–E).

Starvation leads to progressive cell death in *P. bursaria*. Although exact counts were not performed, visual inspection indicated a gradual decline, with approximately 10% of cells remaining on the final day. The images illustrate the typical morphology of surviving cells, where the algal area decreased, but the mitochondrial density remained largely unchanged.

### Changes in mitochondria and cell area of alga-free *P. bursaria* under DD conditions culture

**Fig. 4:**
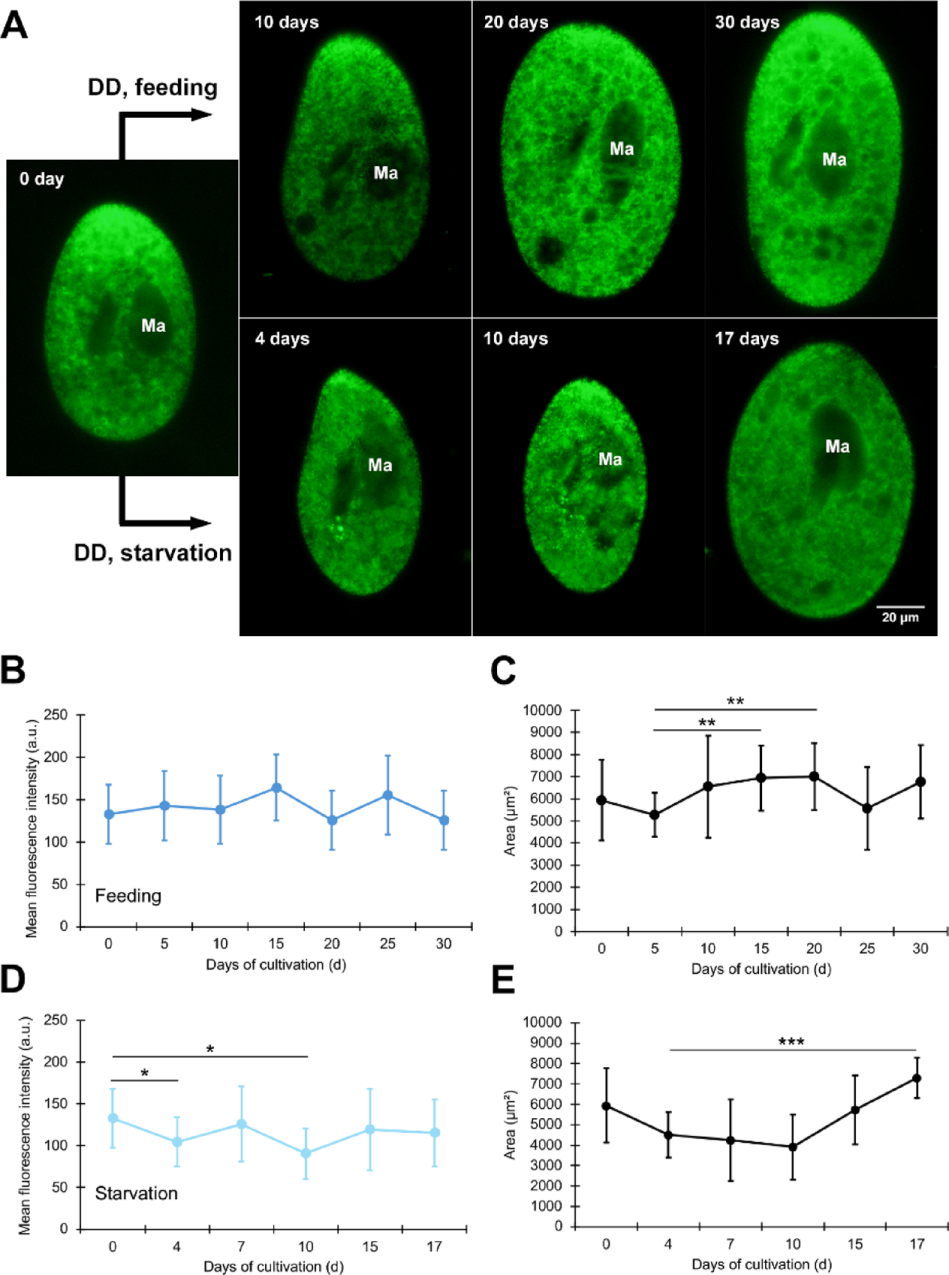
Host mitochondrial fluorescence and cell area in alga-free *P. bursaria* under constant darkness (DD). **(A)** Representative fluorescence micrographs: day 0 (middle panel), DD/feeding (upper panels), and DD/starvation (lower panels). Scale bar = 20 μm. **(B–E)** Quantitative analyses: **(B)** Mean mitochondrial fluorescence intensity (a.u.) under feeding. **(C)** Mean cell area (µm²) under feeding conditions. **(D)** Mean mitochondrial fluorescence intensity (a.u.) under starvation. **(E)** Mean cell area (µm²) under starvation. Error bars = mean ± SD. The raw data used to generate Fig. 1 are provided in Supplementary S3, and the complete statistics are provided in Supplementary S4. Asterisks denote Bonferroni-adjusted- significance: *P* < 0.05 (*), *P* < 0.01 (**), *P* < 0.001 (***).

Figure 4A shows representative fluorescence micrographs, and panels B–E provide quantitative analyses corresponding to these images. Figure 4A shows a micrograph depicting the mitochondrial fluorescence of alga-free *P. bursaria* under the DD conditions. In the feeding group (Fig. 4A, upper panels), mitochondrial fluorescence showed almost no change. In the starvation group (Fig. 4A, lower panels), mitochondrial fluorescence was slightly higher on day 10.

The mean fluorescence intensity (a.u.) of the mitochondria in the feeding group of alga-free *P. bursaria* is shown in Fig. 4B. Variations were observed between days 15 and 30 after the initiation of incubation under DD conditions. In the Kruskal–Wallis (omnibus) test, time-course differences reached significance, but no Bonferroni-adjusted post hoc comparison (including day 0 vs. day 15/30) remained significant. Fluorescence micrographs of 30 cells on day 0, 20 cells on day 5, 21 cells on day 10, 15 cells on day 15, 12 cells on day 20, 17 cells on day 25, and 12 cells on day 30 were analyzed.

The mean cell area (µm²) in the feeding group is shown in Fig. 4C. The mean area was generally high for all the data points. One-way - ANOVA (assumptions met; Levene *p* = 0.065) indicated significant differences (η² = 0.117), and Bonferroni-adjusted post hoc tests identified day 5 vs. day 15 and day 5 vs. day 20 as significant; day 0 vs. day 30 remained non-significant. The cell counts (n) used in Fig. 4C are identical to those in Fig. 4B.

The mean fluorescence intensity (a.u.) of mitochondria in the starvation group of alga-free *P. bursaria* is shown in Fig. 4D, showing an omnibus Kruskal–Wallis difference with small effect; Bonferroni-adjusted post hoc tests confirmed day 0 vs. day 4 and day 0 vs. day 10 as significant. However, no significant difference was detected between the results on days 0 and 17 (*P* = 0.125). Fluorescence microscopy images of 30 cells on day 0, 30 cells on day 4, 18 cells on day 7, 16 cells on day 10, 19 cells on day 15, and 10 cells on day 17 were analyzed.

The mean cell area (µm²) in the starvation group is shown in Fig. 4E. Kruskal–Wallis indicated significant differences with moderate to large effect (ε² = 0.282); Bonferroni-adjusted post hoc tests confirmed day 4 vs. day 17, day 0 vs. day 10, day 10 vs. day 17, day 0 vs. day 7, day 10 vs. day 15, day 7 vs. day 17, and day 0 vs. day 4 as significant. The cell counts (n) used in Fig. 4E are identical to those in Fig. 4D.

Conclusions were based on the quantified means across biological replicates (Fig. 4B–E).

Cells without symbiotic algae were markedly more vulnerable to starvation, showing a rapid decline in survival compared with algae-bearing cells. By the final day, approximately 10% of the initial population of the initial population remained. The images depict the typical morphologies of the surviving cells.

## Discussion

This study examined the response of host mitochondria and cell morphology to symbiont reduction under constant darkness with feeding or starvation conditions. Contrary to expectations, mitochondrial density did not increase as algal numbers declined; instead, nutrient availability was the key factor in maintaining mitochondrial levels. Feeding preserved both cell area and mitochondrial fluorescence despite gradual algal loss, whereas starvation led to marked cell shrinkage and partial mitochondrial decline in algal-free cells. These findings provide new insights into organelle-level responses to symbiont loss and highlight the mechanisms that support the resilience of endosymbiotic systems under environmental stress.

MitoBright was used as a structural marker for mitochondria. This dye provides strong fluorescence and long-term retention but does not report the membrane potential (Δψm), ensuring that our measurements reflect mitochondrial distribution rather than functional status. Accordingly, we interpret MitoBright signals as reporting mitochondrial amount/shape/distribution, and our analyses emphasize density and spatial patterns rather than per-organelle- functional intensity. In addition, MitoBright dyes show higher fluorescence than conventional chloromethyl-containing mitochondrial dyes, supporting their use as robust structural markers in our imaging (https://www.dojindo.com/manual/MT10_MT11_MT12/). Several studies utilizing MitoBright dyes have been reported and adapted to mammalian cells (from the manufacturer’s HP, https://dojindo.co.jp/products/MT10/) and yeast^46^. However, to our knowledge, there have been no reports of MitoBright implementation in protists, including *P. bursaria*. MitoBright dyes can stain cells after PFA fixation. MitoBright signals are retained after post-stain PFA fixation; however, when *P. bursaria* was pre-fixed with 4% PFA prior to staining, mitochondria did not appear as granular structures and fluorescence was diffuse throughout the cell (Ishii and Kodama, data not shown). Live cells were imaged after the transient suppression of ciliary beating with NiCl₂ to prevent dye uptake into DVs. This treatment did not alter mitochondrial morphology, confirming the validity of our imaging methods. The staining patterns were consistent with indirect immunofluorescence using monoclonal antibodies against *P. bursaria* mitochondria^24^, indicating that MitoBright is suitable for investigating mitochondria in *P. bursaria*. In *P. bursaria*, each symbiotic alga is enclosed by a PV, whose membrane adheres to the host mitochondria and anchors the symbiont beneath the host cortex, as described in the Introduction. This arrangement likely limits dye entry into the PV lumen and algal cytoplasm; accordingly, no MitoBright signal was detected within algal mitochondria in our fluorescence images. The endosymbiont is primarily visualized via chlorophyll autofluorescence, whereas MitoBright labels the host mitochondria, and the two signals are spectrally separated. As reported by Kodama and Fujishima (2022)^24^, another commercial fluorescent dye, MitoTracker Green (Molecular Probes, Eugene, OR, USA) can visualize mitochondria in *P. bursaria*; however, specificity is hindered by formation of DVs that ingest dyes, because *Paramecium* spp. can take up non-food substances including fluorescent dyes via the cytopharynx. This nonspecific fluorescence affects quantification. In this study, ciliary movement was first inhibited with NiCl₂ and the cells were then stained with MitoBright. Because ciliary beating is crucial at the initial stage of DV formation^47^, this sequence suppressed phagocytosis, resolving the MitoTracker Green issue. Reversing the order (first staining, then NiCl₂ treatment) resulted in DVs containing MitoBright (Asari and Kodama, unpublished). This order-specific treatment is mechanistically significant and may be applicable to other mitochondrial dyes in future studies.

**Fig. 5:**
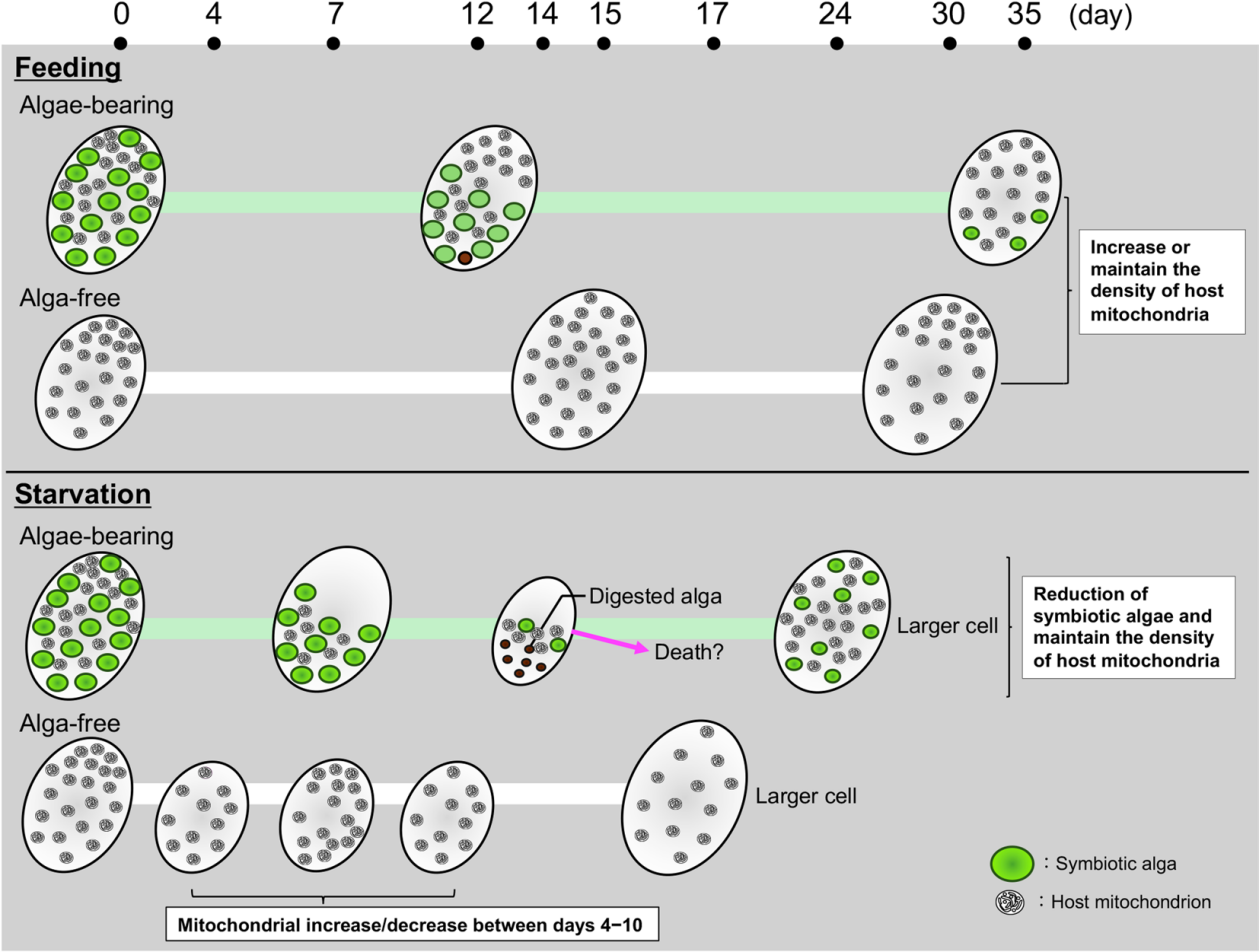
Schematic representation summarizing the results of the study. Panels illustrate feeding (upper) and starvation (lower) conditions across 0–35 d. In algae-bearing *P. bursaria* under DD/feeding, symbiotic algae gradually decreased from the anterior region after approximately 12 d and were rarely observed by day 35, whereas the host cell area remained high and mitochondrial distribution was maintained. Under DD/starvation, symbiotic algae declined earlier (approximately day 7), the host cell area contracted markedly by day 14, and mitochondrial fluorescence weakened around day 8, but it was not significant and was maintained thereafter. In alga-free cells under feeding, mitochondrial fluorescence showed a slight increase by day 15, and the cell area remained stable. Under starvation, alga-free cells exhibited shrinkage within 4 d, followed by partial recovery and increased mitochondrial fluorescence by day 15. This schematic is based on representative cells; the cell sizes are not drawn to scale. Green circles = symbiotic algae; gray symbols = host mitochondria.

Figure 5 summarizes the key patterns observed under constant darkness (DD). Starvation reduced cell area in both algae-bearing and alga-free cells; mitochondrial distribution in algae-bearing cells was preserved, whereas alga-free cells showed a transient decline followed by recovery. Under feeding, mitochondrial distribution was maintained in both states, even though symbiotic algae in the algae-bearing cells gradually decreased. Thus, nutrient availability mitigates shrinkage and prevents the sustained loss of mitochondria. Kornmann et al. (2009)^48^ identified a protein complex termed ERMES (ER-mitochondria encounter structure). The primary function of ERMES is to serve as a mechanical tether between the ER and mitochondria, facilitating communication and metabolite exchange between these organelles. This complex is crucial for efficient phospholipid exchange between the ER and the mitochondria. *Paramecium* mitochondria contain voltage-dependent anion-selective channels (VDAC) in their outer membranes, similar to those found in animal, fungal, and plant mitochondria^49^. This finding suggests a conserved regulatory mechanism for mitochondrial function across various eukaryotic lineages. We hypothesized that under feeding conditions, the host mitochondrial density could be maintained, and consequently, the cell area could be preserved owing to the stability of the lipid supply. In the absence of feeding, the area of the host cells decreased from early to mid-dark incubation but subsequently increased. A plausible interpretation of this result is that the shrunken cells died due to starvation during the intermediate phase of the experiment, and their cellular components were subsequently utilized by the surviving cells, resulting in the ultimate persistence of only the elongated and larger surviving cells.

In the starvation group of algae-bearing *P. bursaria*, symbiotic algae were significantly reduced on day 7 (Fig. 1C), and the cell area was markedly reduced on day 14 (Fig. 1E). In the feeding group, a significant decrease in symbiotic algae was observed from day 12. In addition, the cell area was reduced on day 20 (Fig. 1C). In the starvation group, the abundance of endosymbiotic algae declined, with features consistent with algal digestion/autolysis, a process that supplies nutrients to the host under starvation or constant darkness^34^. Nevertheless, the host cell area decreased, indicating that such symbiont-derived- nutrients were insufficient to maintain the cell area under our conditions. As described in the Introduction, previous investigations employing DD conditions and starvation culture indicated that *P. bursaria* may metabolize algae to acquire nutrients, thereby facilitating trichocyst synthesis^34^. This observation suggests that *P. bursaria* possesses alternative survival mechanisms, such as utilizing nutrients derived from the digestion of symbiotic algae for trichocyst synthesis rather than host cell growth. Conversely, the feeding group exhibited a reduction in symbiotic algae, despite not being subjected to starvation conditions. This phenomenon occurs because lysosomes containing host digestive enzymes fuse with the PV membrane surrounding symbiotic algae under DD conditions, leading to a decrease in the population of symbiotic algae^50^. Subsequently, research has demonstrated that *Chlorella* sp., when incubated for 24 h under DD conditions, exhibit a loss of digestive enzyme resistance within the host DVs^51^.

Symbiotic algae compete with host trichocysts and mitochondria for adhesion sites immediately beneath the host cell surface. Therefore, it has been reported that an increase in the number of symbiotic algae results in a decrease in the number of host trichocysts and mitochondria, whereas a decrease in the number of symbiotic algae leads to an increase in their number^20^. However, the effects of host food bacteria on these phenomena have not yet been investigated. In this study, we hypothesized that when algae-bearing *P. bursaria* were subjected to DD/starvation conditions to induce algal digestion, the density of host mitochondria would increase as the density of symbiotic algae decreased, analogous to the trichocyst phenomenon described previously. However, contrary to expectations, the density of host mitochondria did not increase as the density of symbiotic algae decreased (Figs. 1D and 3D). One interpretation of this result is that it may be attributed to the strong binding of host mitochondria to the PV membrane surrounding the symbiotic algae. It has been hypothesized that symbiotic algae and host mitochondria interact synergistically through these bonds^37^. Consequently, a decrease in symbiotic algae could potentially lead to impaired energy exchange and a corresponding reduction in the host mitochondrial density. This hypothesis requires further investigation.

Under feeding, the mean fluorescence intensity of host mitochondria did not decrease in algae-bearing *P. bursaria* and remained stable, with only a slight upward tendency relative to starvation (Fig. 3B). Because the omnibus tests were not significant and MitoBright reports intensity rather than absolute organelle number, these data indicate the maintenance of mitochondrial density rather than proliferation. Feeding, together with nutrients from algal digestion, likely prevents the decline observed under starvation and helps maintain mitochondrial density, whereas symbiont numbers fall.

The following studies provide evidence of a relationship between the mitochondria and symbiotic algae of *P. bursaria*. Symbiotic algae provide maltose, a photosynthetic product, to the host^1,2^. To facilitate maltose transfer to the host, a hypothetical maltose transporter may be localized in the symbiotic algal plasma membrane^52^. Darkness suppresses light-induced- maltose release from the endosymbiont^53^, and the maltose exporter is proton-gradient-driven- rather than ATP-dependent^54^. No direct causal link is implied; host mitochondrial density is analyzed independently and reflects symbiosis status (lower in algae-bearing cells)^24^. Prolonged darkness can promote symbiont digestion/autolysis^34^, which may indirectly shift cells toward an algal-free state with a higher mitochondrial density^24^.

In the context of our data, on early experimental observation days, we observed a change in the cell area in algae-bearing cells, and a reduction in the density of mitochondria in alga-free cells under DD (Fig. 5). The production of reactive oxygen species (ROS) by mammalian mitochondria is important because it underlies oxidative damage in many pathologies and contributes to retrograde redox signaling from the organelle to the cytosol and nucleus reactive oxygen species^55^. In *P. bursaria*, oxidative stress precipitates a decrease in the photosynthetic products provided to the host, digestion of the symbiont, and destruction of a stable symbiotic relationship^56^. In a previous study, reduced mitochondrial function and elevated expression of antioxidant enzyme genes were reported in the symbiotic algal-bearing *Hydra viridissima* and coral *Acropora digitifera*^57^. Furthermore, glutathione S-transferase (GST) genes are downregulated in algae-bearing *P. bursaria* relative to alga-free cells, and GST enzymes protect cells from oxidative stress^58^. Light is a major environmental regulator of photosynthetic gene expression in higher plants, and its absence (i.e., darkness) leads to the transcriptional downregulation of key genes involved in photosynthesis and redox signaling^59^. These observations suggest that oxidative stress and antioxidant responses may underlie the phenotypic changes observed in constant darkness. Future studies should quantify antioxidant enzyme transcripts to test this hypothesis. Accordingly, the targeted quantification of antioxidant enzyme transcripts in DD-cultured *P. bursaria* will be tested in future studies.

Studying the association between symbionts and host mitochondria provides valuable insights into the complex interactions underlying symbiotic relationships and their effects on host physiology. The intimate connection between the host *P. bursaria* mitochondria and symbionts suggests a potential mechanism for energy exchange and metabolic integration^37^. This structural modification of host organelles may play a crucial role in facilitating beneficial symbiosis. Furthermore, research on cnidarian-dinoflagellate symbiosis has revealed that thermal stress can lead to host mitochondrial degradation, independent of symbiont deterioration^60^. This finding highlights the importance of understanding host responses to environmental stressors and their impact on symbiotic relationships. Interestingly, the study of mitochondrial and chloroplast genomes led to the CORR hypothesis (co-location for redox regulation), which proposes that certain genes are retained in these organelles to facilitate rapid and direct regulatory coupling^61^. This concept could potentially extend to symbiont-host interactions, providing a framework for understanding the molecular dialogue between partners. In conclusion, this study demonstrates that the mitochondrial density in *P. bursaria* does not increase as symbiotic algae decline under constant darkness. Feeding preserved both cell area and mitochondrial fluorescence despite gradual algal loss, whereas starvation caused marked shrinkage and partial mitochondrial decline in the alga-free cells. Future studies should investigate the molecular basis of these responses, including antioxidant pathways and PV-mitochondrial interactions, to clarify how mutualistic associations persist under changing conditions.

## Supplementary Information

Below is the link to the electronic supplementary material.

Supplementary Material 1

Supplementary Material 2

Supplementary Material 3

Supplementary Material 4

## Data Availability

The datasets used in this study are available in the Supplementary Materials.
